# Temporomandibular joint steroid injections in patients with juvenile idiopathic arthritis: an observational pilot study on the long-term effect on signs and symptoms

**DOI:** 10.1186/s12969-015-0060-6

**Published:** 2015-12-21

**Authors:** Peter Stoustrup, Kasper Dahl Kristensen, Annelise Küseler, Thomas Klit Pedersen, Troels Herlin

**Affiliations:** Section of Orthodontics, Aarhus University, Vennelyst Boulevard 9, DK-8000 Aarhus C, Denmark; Specialist Oral Health Center for Western Norway, Stavanger, Rogaland Norway; Department of Oral and Maxillofacial Surgery, Aarhus University Hospital, Aarhus, Denmark; Department of Pediatrics, Aarhus University Hospital Skejby, Aarhus N, Denmark

**Keywords:** Temporomandibular joint, Orofacial pain, Juvenile arthritis, Intra articular, Steroid

## Abstract

**Background:**

Temporomandibular joint (TMJ) inflammation in patients with juvenile idiopathic arthritis (JIA) can lead to orofacial pain and malfunction of the TMJ. Intra-articular corticosteroid injections (IACI) have been suggested as a treatment modality against TMJ arthritis-related orofacial signs and symptoms. However, knowledge of the effect-durability of these injections remains unanswered. The aim of this pilot study was to evaluate the short and long-term effects of IACI on orofacial symptoms in a prospective observational study based on pre-specified clinical examination standards.

**Methods:**

Thirteen patients with JIA and arthritis-related orofacial signs and symptoms were included in this prospective pilot study (median 17.2 years, IQR 15–18.4 years). All patients received TMJ IACI (11 bilateral and two unilateral) due to an insufficient response to previous pain-management treatments. Three standardized clinical examinations were carried out: T1 prior to treatment, T2 short-term follow-up (mean 34 days post-treatment), T3 long-term follow-up (mean 333 days post-treatment).

**Results:**

Significant pain reduction was observed at the short-term follow-up (T2). Resolution of orofacial pain after IACI was a rare finding at T2. Generally, the pain significantly worsened between T2 and T3 examinations. The reported pain levels rose between T2 and T3 indicating a loss of effect of the IACI at the long-term follow-up examination (T3). Non-significant improvements in TMJ mobility were observed at T2 and T3.

**Conclusion:**

Our results suggest a palliative (not curative) effect of IACI for TMJ arthritis-related orofacial symptoms in patients with long-term orofacial pain complaints. The short-term improvements in signs and symptoms were partly resolved at the long-term follow-up.

## Background

In patients with juvenile idiopathic arthritis (JIA), temporomandibular joint (TMJ) involvement may interfere with craniomandibular joint and muscle function [1, 2]. TMJ pain and functional impairment may not necessarily be present in patients with TMJ arthritis [3, 4]. However, when present, the main complaints associated with TMJ arthritis are reduced maximal opening capacity, orofacial pain during TMJ function, fatigue of the jaws, and TMJ crepitation [1, 2, 5]. In some patients the TMJ arthritis-related orofacial pain develops into a more chronic stage which may have a significant impact on the quality of life of the individual patient [6]. It is still unknown whether orofacial pain origins from the actual inflammation or from a TMJ dysfunction preceding morphological erosive changes of the joint components.

The current limited level of evidence suggests potential beneficial properties of intra-articular corticosteroid injections (IACI) in patients with JIA and TMJ arthritis-related orofacial symptoms. However, knowledge of the durability of the effect of these injections needs to be elucidated. In addition, knowledge of the long-term impact of IACI on mandibular growth is unavailable when this treatment modality is administered in growing patients [7].

The aim of the present study was to conduct a pilot study to evaluate the short-term and long-term effects of a single IACI against TMJ arthritis-related orofacial signs and symptoms in a prospective, observational, intention-to-treat-analysis based on pre-specified clinical examination standards.

## Methods

Thirteen patients diagnosed with JIA according to the ILAR criteria [8] were included in the study (all females, median 17.2 years, IQR 15–18.4 years). The included patients represent all JIA patients consecutively referred for treatment with IACI in the TMJ between February 2011 and July 2012 at the Section of Orthodontics, Aarhus University. The patients had a diagnosis of TMJ arthritis based on clinical and radiological findings (cone-beam): At the time of referral all included individuals had findings indicating previous/present TMJ arthritis such as dentofacial growth disturbances and obvious abnormal radiological TMJ findings (degeneration/flattening) in addition to the presence of clinical symptoms. The JIA subtypes are described in Table 1. The indication of referral to IACI treatment was a long-standing history of refractory TMJ arthritis-related orofacial symptoms with an insufficient response to previous pain-management treatments (oral splints, physiotherapy, NSAIDs, MTX and/or biologics). The decision of IACI therapy was based on the following parameters; I) A patient history of severe refractory TMJ arthritis-related orofacial pain, II) functional and/or osseous indications of TMJ arthritis, III) insufficient response to previous treatment. The candidates had all received a cone-beam computerized tomography before the treatment with IACI for the assessment of osseous degenerations; no contrast-enhanced MRIs had been conducted. All patients received non-imaging guided IACI with triamcinolone hexacetonide (20 mg/injection) based on a standardized injection protocol by an experienced and trained operator (TKP). Eleven patients received bilateral TMJ injections and two had unilateral injections. Throughout the study, each patient received three standardized clinical examinations; prior to treatment (T1), at the clinical short-term follow-up examination defined as T2 (34 days post-treatment, range: 7–58 days), and an additional long-term post-treatment examination defined as T3 (mean 333 days post-treatment, range 190–600 days). The standardized examination consisted of a pain-questionnaire completed by the patient and a clinical examination completed by the treating orthodontist. The treatment modality of TMJ IACI in patients with JIA complies with the rules of the Danish Health and Medicines Agency for clinical pediatric rheumatology. The Danish Data Protection Agency approved handling of the confidential data (No.1-16-02-272-14), and all included patients approved the use of the patient files and gave consent to publish the material in accordance with the rules of the Danish Health and Medicines Agency. Outcome assessment was based on the following pain variables:

**Table 1:** Clinical characteristics of 13 patients with JIA with long-term symptomatic TMJ arthritis

Cohort characteristics	*n* = 13
Females, number (percentage)	13 (100 %)
Median age, years (IQR, years)	17 (15–18.4)^a^
TMJ involvement, individuals	
Unilateral	2
Bilateral	11
JIA Diagnosis, individuals	
Oligoarticular extended	3
Oligoarticular persistent	1
Polyarticular	8
Systemic	1
Medication	
NSAID	5
Paracetamol	0
Hydroxychloroquin	1
Methotrexate	5
Biologics (anti-TNF)	9
No medication	1
Combination of 2 to 3 drugs	4
Combination of 4 drugs or more	2
Disease duration, years^b^	
< 1	0
1< 3	1
3–5	2
> 5	10
TMJ involvement, years^c^	
< 1	2
1–3	1
3–5	1
> 5	9

^a^This is the mean age and standard deviation of the 12 patients excluded the outlying patient with the age of 34 years.^b^ Time since JIA diagnosis. ^c^Time since TMJ arthritis diagnosis. *TMJ* temporomandibular joint

### Pain-frequency

Patients were asked to report the frequency of their orofacial symptoms on a 5-point Likert scale: 0) Never pain; 1) pain less than once a week; 2) pain 1–3 times a week; 3) pain 4–6 times a week; 4) pain several times a day; and 5) pain all the time.

### Pain intensity

The patients were asked to assess the average orofacial pain intensity on a non-verbal visual analogue scale (VAS) of 100 mm, where the left extreme represents “no pain” and the right extreme represents the “worst imaginable pain”.

### Pain-index

A composite variable combining the aspects of orofacial pain frequency and intensity; calculated as the pain frequency multiplied by the pain intensity, with a score range between 0 and 500. This variable was used to calculate the relative change in pain between the pre-treatment levels and the short and long-term follow-up pain levels.

Secondary outcome variables describing changes in TMJ mobility were measured in accordance with a previously described standardized examination protocol [9].

### Maximal incisal opening

The maximal unassisted incisal opening capacity was measured inter-incisally with a metallic ruler positioned on the incisal edge of the lower incisors. The inter-incisal vertical overlap was accounted for in the measurement.

### Laterotrusion

The maximal unassisted lateral movement of the lower jaw from the most left position to the most right position measured in mm.

### Protrusion

The maximal unassisted forward movement of the lower jaw measured in mm.

#### Statistics

After the data were tested for normal distribution general differences in pain variables and functional variables between T1, T2 and T3 were assessed by ANOVA tests with paired Student’s *t*-tests serving as post-ANOVA tests. Post-Anova tests were only performed in outcome variables where a statistical significant difference was observed in the primary ANOVA test. Changes in the categorical data concerning pain frequency were statistically analyzed using a Kruskal Wallis Rank-sum test with Wilcoxon matched-paired rank tests serving as post-tests. A *p* value of less than 0.05 was considered significant.

## Results

Radiological signs of osseous TMJ degenerations were observed in all TMJs receiving IACI. Patient characteristics are depicted in Table 1. Two patients had their treatment changed during the observation: One patient had a change in the medication between T1 and T2; systemic anti-TNF-α (Enbrel) treatment was added to the treatment of methotrexate after experiencing no effect of the bilateral TMJ IACIs. Another patient received additional bilateral TMJ IACIs between T2 and T3 due to a lack of effect of the initial IACI treatment. Both of these patients were included in the final analysis based on a last-observation-carried-forward strategy. No adverse reactions were observed directly in relation to the IACI procedure.

**Fig. 1:**
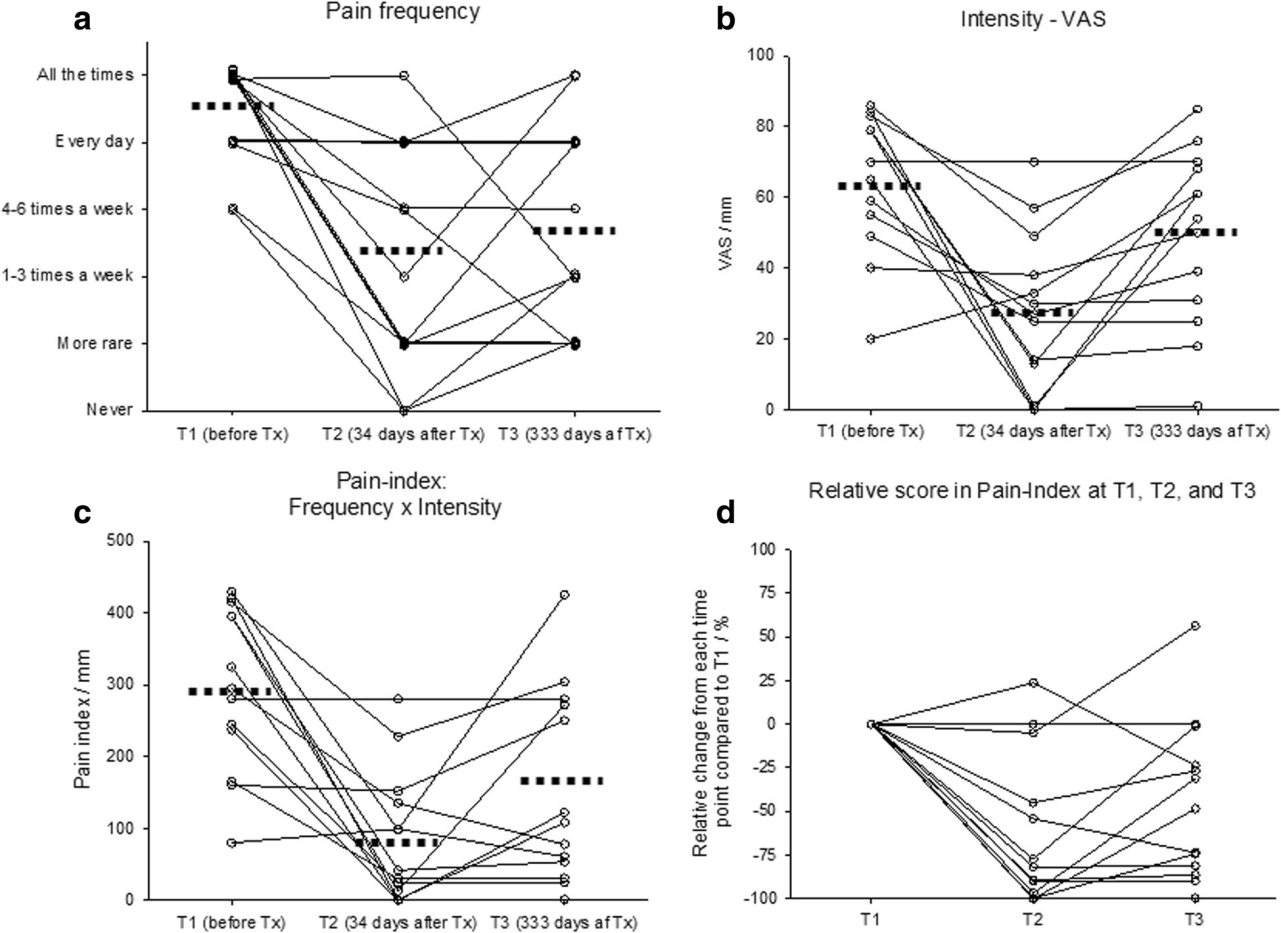
Pain related outcomes. Changes in outcome variables related to pain before and after corticosteroid injection of the TMJ in 13 patients with JIA. Dotted lines indicate the mean values at each point of time. **a** changes in pain frequency, **b** changes in pain intensity, **c** changes in painindex (frequency x intensity), **d** Relative pain-scores at different timepoints

Pre- and post-treatment pain and functional values are depicted in Table 2 and Fig. 1. The Kruskal Wallis and the ANOVA tests revealed statistical intra-group differences between the three time-points in relation to all three pain outcome variables. Further intra-group differences were revealed during post-testing and these are reported below. No general primary significant differences were observed for any of the functional outcome variables.

**Table 2:** Changes in outcome variables

	T1-Before treatment (SD)	T2-Short-term follow-up (SD)	T3-Long-term follow-up (SD)	Diff. T2-T1/ (SD)	Diff. T3-T1/ (SD)	Diff. T3- T2/ (SD)	ANOVA *p*-value^a^	Post-tests^b^
Pain								
Pain frequency (1–5)	4.5¤	2.3¤	2.7¤	2.2	1.8	0.4	0.0001^d^	T1 > T2 = T3^e^
Pain intensity (VAS 100 mm)	65.2 (19.9)	27.5 (22.2)	49.3 (24.8)	−37.8 (31.4)	−16.1 (27.7)	21.7 (22.7)	0.0006	T1 > T2 < T3, T1 = T3,
Pain index (Frequency x intensity)	295.5 (114.2)	84.9 (91.7)	154.7 (134.9)	−210.7 (149.1)	−140.8 (136.4)	69.8 (114.4)	0.0002	T1 > T2 < T3, T1 > T3,
Function								
Mouth opening (mm)	35.5 (7.1)	40.3 (5.8)	39.3 (6.5)	4.9 (5)	3.9 (7.5)	−1 (6.5)	0.24	^c^
Laterotrusion (mm)	12.3 (4.8)	14.9 (2.9)	15.8 (5.4)	2.6 (3.4)	3.5 (3.5)	0.9 (4.2)	0.13	^c^
Protrusion (mm)	5.4 (1.5)	6.3 (1.7)	7.2 (2.9)	0.9 (2.1)	1.8 (2.7)	0.9 (2.9)	0.09	^c^

^a^ANOVA is used if nothing else i stated. ^b^Students paired *T*-test is used if nothing else is stated. A significant difference (*p* < 0.05) is indicated with”<” or “>”. ^c^Post-Anova tests were only performed in outcome variables were a statistical significant difference was observed in the primary ANOVA test. ^d^Kruskal Wallis Ranksum test. ^e^Wilcoxon matched paired Rank test. ¤ A mean value has been calculated despite of the categorical nature of this variable

### Pain-variables

High pre-treatment pain levels were seen at T1 with a mean pain intensity of 62.7 (VAS scale 0–100 mm) and a reported average pain frequency of “several times a day”. At the short-term follow-up (T2) pain-frequency, pain-intensity and pain-index were all significantly reduced when compared to the pre-treatment T1 levels. However, at the long-term T3 follow-up only the pain-frequency remained significantly reduced compared to the pre-treatment T1 level. The pain-intensity and also the pain-index variables significantly worsened between T2 and T3 as illustrated in Table 2; especially patients with high pre-treatment pain levels had a tendency to worsen between T2 and T3.

### Relative pain-index reduction

Pain cessation was rare at the follow-ups and was only experienced in three patients at the short-term follow-up (T2) and in one patient at the long-term T3 follow-up (Fig. 1d). At T2, eleven patients reported a relative pain-index reduction when compared to pre-treatment levels; nine patients (69 %) had experienced a reduction of more than 50 per cent at T2 (Fig. 1d). Between the short- and the long-term examination 7 patients experienced an aggravation of the reported pain-index levels potentially indicating a reduction in the effect of the IACI treatment (Fig. 1d). However, six patients reported a relative T3 pain-index reduction of more than 50 per cent compared to the pre-treatment T1 levels.

### TMJ mobility

**Fig. 2:**
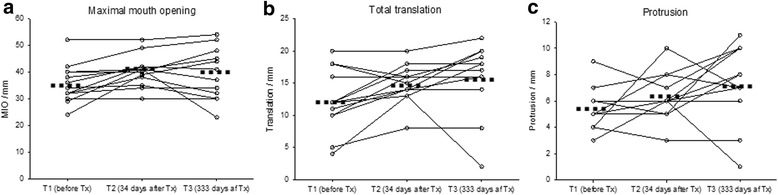
Outcomes in TMJ mobility. Changes in outcome variables related to TMJ mobility before and after corticosteroid injection of the TMJ in 13 patients with JIA. Dotted lines indicate the mean values at each point of time. **a** changes in maximal mouth opening, **b** changes in total laterotrusion, **c** changes in protrusion

The ANOVA test documented no significant intra-group changes in any of the outcome variables reflecting TMJ mobility (Table 2). Despite this finding, Fig. 2 reveal great inter-patient variation in the functional changes after IACI and at the short-term examination clinical relevant improvements are seen in some patient.

## Discussion

We observed a significant pain-level reduction in the majority of patients at the short-term follow-up evaluation pointing towards a beneficial short-term effect of IACI. Although statistical significant symptomatic improvements of TMJ arthritis were observed, this pilot study shows a considerable inter-patient variation in the response to IACI treatment and total resolution of orofacial pain was a rare finding. Between the short-term (T2) and the long-term (T3) follow-up, exacerbation of pain-levels was seen in seven out of thirteen patients suggesting a loss of effect of the IACI at the long-term follow-up examination for some of the patients. Aggravation of pain between the short-term and the long-term follow-up was most pronounced in patients with high T1 pre-treatment pain levels. This finding is somewhat in conflict with previous reports on IACI for the treatment of TMJ arthritis-related signs and symptoms where a better response has been reported from IACI TMJ treatment [10–12]. This difference in the response to the treatment in our study could be explained by the included group of patients where a majority of the adolescent and adult JIA patients had a long-term history of pain complaints and an insufficient response to previous orofacial pain management strategies. Previous studies have included JIA patients of younger age and with shorter duration of TMJ arthritis [10–12]. However, when assessing our results it still worth to notice that six out of the thirteen patients had a relative pain-index reduction of more than 50 per cent at the long-term follow-up compared to the pre-treatment levels; especially, reduction pain frequency was observed between T1 and T3. This is a positive finding related to this group of patients with persistent and long-term pain issues failing other pain treatment strategies. However, the study design does not allow us to assess if this is due to fluctuation of orofacial symptoms or the real effect of the treatment with IACI since no control group is included.

The present group of patients gives the impression of a pain problem not only related to TMJ arthritis but also to the morphological changes within the TMJ caused by the arthritis. The results presented in this study, therefore, are probably not generalizable to a whole JIA population since the present group of patients is a unique group of patients suffering from refractory TMJ arthritis-related pain. However, from a clinical point of view our results contribute with new knowledge since it illustrates that achievement of an acceptable treatment result in this group of patients is indeed a very challenging task. Secondly, this is the first longitudinal study to address the treatment effect with IACI in this specific patient group with refractory orofacial symptoms. It is possible that a population-based group of JIA patients with TMJ involvements could have a better outcome of IACI in relation to arthritis-induced orofacial pain.

Despite the fact that no significant improvements in TMJ mobility changes were observed throughout the study, minor short-term and long-term improvements were seen after the treatment with IACI. The clinical relevance of the long-term TMJ mobility improvements described is debatable since only short-term improvement in TMJ mobility exceeds the variation within the measurement technique used to assess the outcome variables examined [9]. Our TMJ mobility findings are therefore in line with previous reports on the effect of IACI on mouth opening capacity where only minor improvements, with limited clinical value, have been obtained [10, 13, 14].

The current limited evidence available suggests potential beneficial properties of IACI against TMJ arthritis-related symptoms. At this point no clear scientific evidence substantiates the effect of IACI in terms of improvement in orofacial function [7]. From a clinical point of view, our findings support the present clinical indications for the use of IACI against TMJ arthritis. However, caution should be applied before the use of IACI in younger patients since concerns about the impact of IACI on mandibular growth remain an unanswered and relevant consideration [7]. Another concern in relation to TMJ IACI is the risk of developing intra-articular calcifications which may reduce the TMJ function: A recent publication by Lochbühler et al., observed intra-articular calcifications in 21 percent of the TMJs that received treatment with IACI [15].

Important strengths of this study were the prospective, standardized, intention-to-treat design. This is the first study to use this design within this specific field and treatment modality. Notable limitations were the low number of included patients, the lack of a control group to account for the bias of the placebo-effect, and the selection bias during the referral procedures. Another limitation was the fact that no pre-treatment MRI was performed in any of the patients. However, recent research suggests only a limited association between clinical findings and TMJ MRI findings in patients with JIA [16]. In addition, a recent report calls for a general debate about the MRI-indications for the use of IACI therapy, since MRI contrast-enhancement seems to be a normal finding in the soft tissue and the mandibular condyle of the TMJ in non-arthritic children and adolescents [17].

## Conclusion

In conclusion, our results suggest a palliative (not curative) effect of IACI for TMJ arthritis-related orofacial symptoms in patients with long-term orofacial pain complaints not responding to other pain management strategies. The short-term improvements in signs and symptoms after IACI were partly resolved at the long-term follow-up. Future studies in accordance with evidence based standards, involving more patients, are needed in order to make more solid conclusions. Based on the findings of this pilot-study with TMJ IACI, future, prospective, long-term follow-up studies are planned.
